# Epidermal Growth Factor Receptor (EGFR) Amplification and Survival in Patients With Glioblastoma: A Systematic Review and Meta-Analysis

**DOI:** 10.7759/cureus.113349

**Published:** 2026-07-25

**Authors:** Maria P Fernandez Gomez, William A Florez Perdomo, Guillermo de Jesus Aguirre Vera, Jesus Francisco Saltaren, Luis R Moscote-Salazar, Raja Vadivel KR, Rakesh Mishra, Amit Agrawal, Jose Valerio

**Affiliations:** 1 Department of Neurological Surgery, Latinoamerica Valerio Foundation, Miami, USA; 2 Faculty of Medicine, Surcolombiana University, Neiva, COL; 3 School of Medicine and Health Sciences, Tecnológico de Monterrey "Ignacio A. Santos", Mexico City, MEX; 4 Department of Plastic and Reconstructive Surgery, Hospital de Kennedy, Bogotá, COL; 5 Department of Neurosurgery, Red Latino, Cartagena, COL; 6 Department of Trauma and Emergency Medicine, All India Institute of Medical Sciences, Bhopal, IND; 7 Department of Neurosurgery, All India Institute of Medical Sciences, Bhopal, IND; 8 Department of Neurological Surgery, Palmetto General Hospital, Hialeah, USA

**Keywords:** egfr mutations, genetics of glioblastoma, mgmt gene methylation, overall survival (os), systematic review and meta-analysis

## Abstract

Glioblastoma has been described as the most common and aggressive primary brain neoplasm in adults, with limited survival despite multimodal therapy. The prognostic impact of epidermal growth factor receptor (EGFR) amplification and its interaction with isocitrate dehydrogenase (IDH) mutation and O6-methylguanine-DNA methyltransferase (MGMT) promoter methylation remains unclear. This systematic review and meta-analysis examined how EGFR amplification level and molecular profile affect overall survival (OS) in glioblastoma. Following the Preferred Reporting Items for Systematic Reviews and Meta-Analyses (PRISMA) 2020 guidelines, a systematic review was performed for studies reporting OS stratified by EGFR amplification, MGMT methylation, or IDH mutation. Using a random-effects model, pooled hazard ratios (HRs) with 95% confidence intervals (CIs) were calculated, and I² statistics were employed to assess heterogeneity. Nine studies including 1,766 subjects were analyzed. Low or moderate EGFR amplification showed no OS impact (HR=0.98 (95% CI: 0.88-1.09); HR=0.94 (95% CI: 0.81-1.10)), whereas high amplification predicted worse survival (HR=1.19; 95% CI: 1.14-1.24; p<0.00001). EGFR amplification with MGMT methylation correlated with poorer outcomes (HR=1.38; 95% CI: 1.33-1.44), while EGFR amplification with unmethylated MGMT showed improved survival (HR=0.68; 95% CI: 0.61-0.76). Stratification by IDH status identified EGFR-negative/IDH-mutant tumors as the most favorable group (HR=0.44; 95% CI: 0.29-0.67) and EGFR-positive/IDH-wildtype as the least favorable (HR=1.33; 95% CI: 0.89-2.00). High EGFR amplification independently predicts worse survival in glioblastoma, particularly with MGMT promoter methylation and IDH-wildtype status. Integrating EGFR, MGMT, and IDH profiles refines prognostic assessment and supports personalized therapeutic strategies.

## Introduction and background

Glioblastoma is the most common and aggressive primary malignant brain neoplasm in adults, with a median overall survival (OS) of approximately 12-18 months despite maximal safe resection, radiotherapy, and temozolomide-based chemotherapy [1]. Its marked molecular heterogeneity contributes to treatment resistance, recurrence, and substantial variation in clinical outcome. Although epidermal growth factor receptor (EGFR) amplification is among the most frequent genomic alterations in glioblastoma, its independent prognostic significance has remained inconsistent across published studies. The 2021 World Health Organization Classification of Tumors of the Central Nervous System (WHO CNS5) reorganized adult-type diffuse gliomas into three principal entities: astrocytoma, isocitrate dehydrogenase (IDH)-mutant; oligodendroglioma, IDH-mutant and 1p/19q-codeleted; and glioblastoma, IDH-wildtype [2]. Importantly, the term glioblastoma is now restricted to IDH-wildtype tumors. Neoplasms previously designated as secondary glioblastomas are classified as astrocytoma, IDH-mutant, CNS WHO grade 4. Thus, IDH status is not simply an additional prognostic variable; it defines biologically and diagnostically distinct diseases.

IDH-mutant and IDH-wildtype diffuse gliomas follow different molecular evolutionary pathways and have markedly different clinical behavior. IDH1 and IDH2 normally participate in cellular metabolism; oncogenic IDH mutations alter enzymatic activity and induce broad epigenetic remodeling, thereby establishing a distinct tumor lineage that generally occurs in younger patients and is associated with more favorable survival than IDH-wildtype glioblastoma [3-5]. EGFR amplification shows the opposite distribution: it is enriched in the classical IDH-wildtype glioblastoma phenotype and is uncommon in IDH-mutant diffuse gliomas [3,4]. WHO CNS5 recognizes EGFR amplification, together with specific histologic and molecular criteria, as one of the molecular features that can support the diagnosis of glioblastoma, IDH-wildtype, CNS WHO grade 4 in adult diffuse astrocytic tumors lacking necrosis or microvascular proliferation [2]. Accordingly, IDH mutation and EGFR amplification should not be interpreted as independent, randomly distributed prognostic factors; they are closely linked to different glioma entities.

These biomarkers represent complementary rather than interchangeable biological processes. EGFR is a receptor tyrosine kinase, and amplification increases signaling through the PI3K-AKT-mTOR and RAS-RAF-MEK-ERK pathways, promoting tumor cell proliferation, invasion, angiogenesis, and treatment resistance [5,6]. O6-methylguanine-DNA methyltransferase (MGMT) encodes a DNA repair enzyme that removes alkyl adducts from the O6 position of guanine; promoter methylation reduces MGMT expression and generally increases tumor sensitivity to temozolomide [5]. In practical terms, IDH status defines the tumor entity and its baseline biological and prognostic context, EGFR amplification reflects the activation of mitogenic signaling within that context, and MGMT promoter methylation primarily modifies treatment responsiveness. Their associations with survival should therefore be interpreted jointly rather than as independent and equivalent prognostic variables.

Because EGFR amplification occurs predominantly in IDH-wildtype glioblastoma, observed associations between EGFR amplification and poorer survival should be interpreted within this molecular context rather than assuming that EGFR amplification alone accounts for the prognostic differences. Failure to account for this molecular hierarchy may confound comparisons between EGFR-amplified and EGFR-non-amplified tumors, particularly in historical cohorts classified before integrated molecular diagnosis became standard. The prognostic interpretation of EGFR must also consider MGMT status, because treatment sensitivity may modify observed survival independently of proliferative signaling. On this basis, the present systematic review and meta-analysis evaluated the association between EGFR amplification and OS while considering IDH mutation and MGMT promoter methylation as essential molecular contexts for interpretation.

The transition to integrated molecular classification under WHO CNS5 also presents an important challenge for interpreting historical studies. Many earlier cohorts were diagnosed using predominantly histopathologic criteria and therefore may have included tumors that would now be classified as different molecular entities. Consequently, comparisons across studies spanning different classification eras should be interpreted with caution, as apparent prognostic differences may partly reflect changes in diagnostic definitions rather than true biological differences.

## Review

Methods

Study Design and Reporting

The review was conducted and reported with reference to the Preferred Reporting Items for Systematic Reviews and Meta-Analyses (PRISMA) 2020 statement [7].

Eligibility Criteria

Studies were eligible if they enrolled patients with glioblastoma, evaluated EGFR gene amplification in relation to OS, and reported hazard ratios (HRs) with corresponding confidence intervals or sufficient data to derive time-to-event effect estimates. Studies using predefined categories of EGFR amplification (e.g., amplified vs. non-amplified or other study-specific amplification classifications) were considered eligible. The complete eligibility criteria are summarized in Table 1.

**Table 1:** Inclusion and exclusion criteria used for study selection Historical glioblastoma diagnoses were accepted as originally reported because many eligible studies predated the implementation of the 2021 WHO Classification of Tumors of the Central Nervous System (WHO CNS5). Molecular classification was considered during the interpretation of the findings. EGFR: epidermal growth factor receptor; PCR: polymerase chain reaction; FISH: fluorescence in situ hybridization; HRs: hazard ratios

Category	Criterion
Inclusion	Studies enrolling adults diagnosed with glioblastoma in the original report. Historical diagnoses were accepted. Mixed adult grade 4 glioma cohorts derived from an originally histologic glioblastoma population were retained when their molecular composition could be characterized; such estimates were interpreted as mixed-cohort rather than entity-specific effects
	Studies evaluating the association between EGFR amplification and survival using study-defined amplification categories
	Overall survival was required as the primary outcome. Progression-free survival and extent of resection were extracted when reported as secondary contextual variables
	EGFR amplification assessed using genomic copy-number methods, including PCR, FISH, or other validated molecular technique
	Randomized controlled trials
	Non-randomized clinical trials
	Observational studies (prospective or retrospective cohorts)
	Studies reporting HRs or sufficient data to calculate them
Exclusion	No separable glioblastoma population, did not evaluate EGFR amplification, or no eligible comparator group
	Studies focused on therapeutic efficacy, experimental delivery systems, or unrelated molecular targets without estimating the prognostic association between baseline EGFR amplification and survival
	Studies in which glioblastoma-specific survival data could not be distinguished from other diffuse glioma subtypes
	Case reports or case series
	Reviews, editorials, letters, or non-original research
	Preclinical studies (animal or in vitro studies only)

Rationale for Eligibility Criteria

Eligibility was restricted to original human studies with a directly relevant population, exposure, comparator, and outcome because the review question concerned the clinical prognostic value of EGFR gene amplification rather than EGFR expression, experimental targeting, or tumor biology alone. Studies enrolling patients diagnosed with glioblastoma in the original reports were eligible. When contemporary molecular reassessment identified more than one WHO CNS5 grade 4 entity within an originally histologic glioblastoma cohort, the estimate was retained but interpreted as a mixed-cohort effect rather than as a glioblastoma-specific estimate. A study-defined comparator based on EGFR amplification status or predefined amplification categories was required to permit the estimation of relative prognosis. OS was required for quantitative synthesis because it was the prespecified primary endpoint; progression-free survival and extent of resection were recorded only as secondary contextual variables. Finally, studies had to report an HR with a measure of uncertainty or provide Kaplan-Meier or numerical time-to-event data sufficient to derive a compatible estimate. Randomized trials, non-randomized trials, and prospective or retrospective cohorts were eligible because prognostic biomarker evidence is commonly generated in observational cohorts, provided that the required comparison and survival information were available.

Because many eligible studies predated the implementation of WHO CNS5, historical diagnostic classifications reported by the original investigators were accepted during study selection. Differences between historical and contemporary molecular classification were considered during the interpretation of the findings rather than used as exclusion criteria.

Rationale for Exclusion Criteria

Studies were excluded when they did not isolate a glioblastoma population, did not evaluate EGFR gene amplification, lacked a non-amplified or amplification-level comparator, or did not provide extractable time-to-event survival data. Studies based solely on EGFR protein expression were not pooled because expression and gene copy-number amplification represent related but non-interchangeable measurements. Case reports and small uncontrolled case series were excluded because they are unlikely to provide stable comparative hazard estimates and are especially susceptible to selection bias. Reviews, editorials, and letters were excluded because they do not contribute independent patient data. Animal experiments, in vitro studies, nanoparticle delivery investigations, and cellular therapy studies were excluded because they address mechanism or treatment efficacy rather than the prognostic association between baseline EGFR amplification and survival in a clinical cohort.

Temporal Scope and Molecular Classification Considerations

The search covered database inception through March 2025 without imposing a general publication year restriction. This approach allowed inclusion of both historical and contemporary studies evaluating the association between EGFR amplification and survival in glioblastoma. Nevertheless, older studies were not assumed to be diagnostically interchangeable with contemporary WHO CNS5-defined glioblastoma. Reports published before routine IDH testing and integrated molecular classification may have included tumors that would now be classified as astrocytoma, IDH-mutant, CNS WHO grade 4 rather than glioblastoma, IDH-wildtype [2].

Publication era, availability of IDH and MGMT data, amplification assay, treatment period, and adjustment for confounding were therefore considered during interpretation. Contemporary WHO, The Cancer Genome Atlas (TCGA), and large molecular cohort publications that informed biological and clinical interpretation but did not provide an eligible survival comparison based on EGFR amplification were cited narratively and were not included in the quantitative synthesis.

Historical studies were retained because they contributed clinically relevant evidence regarding EGFR amplification. Given the evolution of glioblastoma classification and differences in molecular reporting across study periods, pooled estimates were interpreted within the clinical and methodological context of the individual studies rather than solely on the basis of the quantitative summary measures.

Information Sources

Cochrane Library, MEDLINE (Ovid), Scopus, Embase (Ovid), PubMed, CINAHL, and Web of Science were searched through March 2025. Searches covered database inception except where a database-specific limit was explicitly included in the strategy. Reference lists were screened by backward and forward citation searching, and supplementary searches were conducted in Google Scholar and ScienceDirect using search terms related to EGFR amplification, glioblastoma, and survival.

Search Strategy

Electronic searches were developed using combinations of controlled vocabulary and free-text terms related to glioblastoma, EGFR gene amplification, and survival (Appendices). Search terms included "glioblastoma", "glioblastoma multiforme", "GBM", "malignant glioma", "EGFR amplification", "epidermal growth factor receptor amplification", "EGFR gene amplification", "EGFR copy-number gain", "EGFR overexpression", "overall survival", "survival", "prognosis", and "mortality". Boolean operators and database-specific indexing terms were adapted for each source. The Scopus search was restricted to publications after 2018. Reference lists of included studies and relevant reviews were screened, and supplementary searches were conducted in Google Scholar and ScienceDirect using combinations of "EGFR amplification", "glioblastoma", and "survival".

Selection Process

Two reviewers independently screened the titles and abstracts of all retrieved records and classified them as eligible, ineligible, or requiring full-text assessment. Full-text reports considered potentially eligible or unclear were then assessed against the prespecified eligibility criteria. Disagreements were resolved through discussion and, when necessary, consultation with a third reviewer. Reasons for exclusion at the full-text stage were documented. No language restrictions were applied during study selection; all studies meeting the eligibility criteria were published in English.

Data Collection

Two reviewers independently extracted data using a piloted standardized form. Extracted variables included author, country, recruitment setting and period, publication year, study design, sample size, participant age, diagnostic classification (histologic and/or molecular, when reported), EGFR assay and amplification threshold, IDH and MGMT status when available, treatment characteristics, duration of follow-up, OS definitions, reported HRs, 95% CIs, and covariates included in adjusted models. For IDH status, studies were classified as molecularly defined IDH-wildtype cohorts, mixed IDH-defined grade 4 glioma cohorts, or historical cohorts without sufficient IDH characterization. When a study reported more than one eligible estimate for the same comparison, the multivariable-adjusted HR with the most complete adjustment for clinically relevant confounders was preferred whenever available. Disagreements were resolved by consensus with a third reviewer.

Assessment of Potentially Overlapping Populations

Potentially duplicate or overlapping cohorts were assessed by comparing author groups, institutions, countries, recruitment periods, eligibility criteria, sample sizes, molecular testing methods, and baseline characteristics across reports. When two publications were judged to describe the same or partially overlapping population and reported the same survival contrast, the prespecified rule was to retain the report with the largest eligible sample, longest follow-up, or most complete adjusted estimate. Distinct non-overlapping subgroups from the same source could be retained when participants were not counted twice within a pooled comparison. Based on the information available in the published reports, no evidence of definite duplicate cohorts was identified among the studies included in the quantitative synthesis.

Risk of Bias and Study Quality Assessment

Two reviewers independently assessed the non-randomized studies using ROBINS-I, adapted to evaluate EGFR amplification as the exposure of interest [8]. The appraisal considered bias due to confounding, participant selection, classification of the exposure, deviations from the exposure definition, missing data, outcome measurement, and selective reporting. The Newcastle-Ottawa Scale was used as a complementary assessment of cohort study quality [9]. Disagreements were resolved by consensus with a third reviewer.

Effect Measures and Derivation of HRs

OS was summarized using HRs and 95% CIs. An HR greater than 1 indicated a higher instantaneous mortality hazard in the EGFR-defined group, whereas an HR below 1 indicated a lower hazard. Study-specific estimates were analyzed on the natural-log scale. When an article reported an HR and its 95% CI, the log HR was calculated as ln(HR), and its standard error was derived as follows: $\text{[ln(upper CI)&minus;ln(lower CI)]/3.92}$.

Directly reported log HRs and standard errors were used without further transformation. When an HR was not directly reported, an estimate was included only when the publication provided sufficient numerical time-to-event information to derive a compatible log HR and standard error. Studies lacking sufficient information to derive a compatible estimate or measure of precision were not quantitatively synthesized. Odds ratios and risk ratios were not combined with HRs. Pooled estimates were back-transformed and presented as HRs with 95% CIs.

Quantitative subgroup analyses examined the EGFR amplification categories reported by the included studies and MGMT promoter methylation status when compatible estimates were available. IDH status was used to characterize molecular cohort composition and diagnostic comparability. Quantitative pooling according to IDH status required directly comparable EGFR-specific HRs within the same IDH-defined tumor entity; when such estimates were unavailable, IDH-related findings were synthesized narratively.

Statistical Methods and Model Selection

Meta-analyses were performed in Review Manager (RevMan, The Cochrane Collaboration, London, England, United Kingdom) using the generic inverse-variance method to pool study-specific log HRs and standard errors. A random-effects model was selected a priori because clinically and methodologically relevant variation was expected across studies in participant age, diagnostic era, molecular classification, EGFR assay and amplification threshold, treatment exposure, follow-up duration, and covariate adjustment.

Between-study heterogeneity was summarized using tau-squared (τ²), Cochran's Q test, and the I² statistic. The I² value was interpreted together with the direction, magnitude, and precision of the study estimates rather than according to an isolated numerical threshold. Statistical significance for pooled effects was defined as a two-sided p-value of <0.05.

Potential sources of heterogeneity were explored through quantitative subgroup analyses according to study-defined EGFR amplification categories and MGMT promoter methylation status when sufficient compatible data were available. IDH-defined cohort composition, assay methods, diagnostic era, treatment characteristics, and covariate adjustment were compared qualitatively. Meta-regression was not performed because no comparison included enough studies to support stable regression coefficients, and relevant study-level covariates were reported inconsistently. Under these conditions, meta-regression would have been underpowered and susceptible to overfitting and ecological bias.

Subgroup Analyses and Statistical Interpretation

Quantitative subgroup analyses according to study-defined EGFR amplification categories and MGMT promoter methylation status were interpreted as exploratory. When a formal test for subgroup differences was available, it was used to assess whether effect estimates differed statistically across molecular strata. A statistically significant pooled estimate in one subgroup and a nonsignificant estimate in another was not considered evidence of interaction unless supported by a formal between-subgroup test.

IDH status was evaluated as an entity-defining molecular classification variable rather than as a directly comparable biomarker subgroup. A quantitative EGFR-by-IDH synthesis required compatible EGFR-specific HRs reported separately within IDH-defined tumor entities. Because molecular ascertainment and reporting differed across diagnostic eras, IDH-related findings were summarized narratively when this requirement was not met.

Because each quantitative subgroup contained a small number of studies and definitions of biomarker status, treatment exposure, and covariate adjustment varied across cohorts, p-values and 95% CIs were interpreted together with effect direction, precision, and heterogeneity. The subgroup analyses were not used to infer causality or establish an independent quantitative ranking of molecular biomarkers.

Assessment of Small-Study Effects and Publication Bias

Funnel plot inspection and Egger's regression test were planned only for pooled comparisons containing at least 10 studies because asymmetry tests have limited power and may be misleading when fewer studies are available. No quantitative comparison in this review met that threshold; therefore, formal assessment of funnel plot asymmetry was not performed. The possibility of publication and selective reporting bias was instead considered qualitatively in light of the comprehensive database search, supplementary searching, outcome availability, and the small number of studies contributing to each synthesis.

Results

Study Selection

A total of 120 records were identified through the database searches. After the removal of 70 duplicates, 50 records underwent title and abstract screening, and 13 reports were assessed in full text. Four reports were excluded after full-text assessment. Layfield et al. [10] evaluated EGFR amplification, protein expression, and prognosis but did not report an extractable HR with a corresponding measure of precision or sufficient compatible time-to-event data to derive one. Pickering et al. [11] was a preclinical nanoparticle drug delivery study without a human prognostic cohort. Begley et al. [12] was a narrative review and therefore did not provide independent comparative patient data. Courot et al. [13] evaluated EGFR-directed natural killer cells in vitro and did not report clinical survival outcomes.

**Figure 1:**
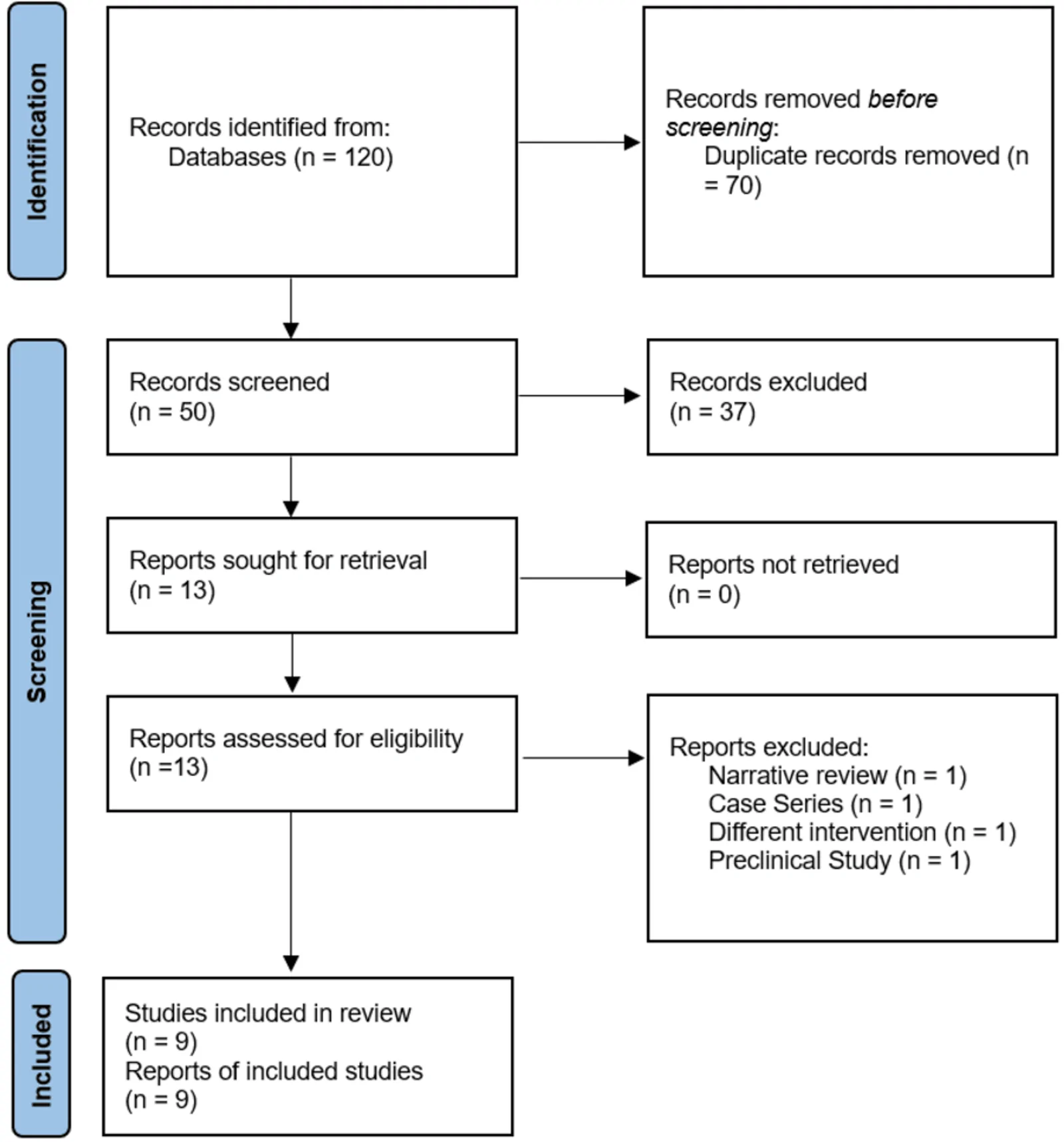
PRISMA flowchart of the study selection process PRISMA: Preferred Reporting Items for Systematic Reviews and Meta-Analyses

Nine cohort studies, one prospective and eight retrospective, met the inclusion criteria and were included in the review [14-22]. The study selection process is shown in Figure 1.

Study Characteristics

The nine included studies were published between 2003 and 2025 and used varying molecular methods and thresholds to assess EGFR amplification [14-22]. Sample size, molecular characterization, treatment context, and diagnostic era varied across studies. Several studies predated routine IDH testing and the integrated WHO classifications introduced in 2016 and 2021. They were retained because they reported eligible survival comparisons according to EGFR amplification status, but their historical diagnostic frameworks were considered an important source of clinical heterogeneity. The characteristics of the included studies are summarized in Table 2.

**Table 2:** Characteristics of the included studies EGFR: epidermal growth factor receptor; MGMT: O6-methylguanine-DNA methyltransferase; IDH: isocitrate dehydrogenase; WHO: World Health Organization; WHO CNS5: World Health Organization Classification of Tumors of the Central Nervous System

Study	Country	Design	Sample size (n)	Molecular variables reported	Cohort note
Shinojima et al. 2003 [22]	Japan	Retrospective cohort	87	EGFR; MGMT	Historical histologically defined glioblastoma cohort; IDH not reported; pre-integrated WHO classification
Quan et al. 2005 [21]	USA	Retrospective cohort	107	EGFR	Historical histologically defined glioblastoma cohort; IDH not reported; pre-integrated WHO classification
Kleinschmidt-DeMasters et al. 2005 [18]	USA	Retrospective cohort	20	EGFR	Historical elderly glioblastoma cohort; IDH not reported; pre-integrated WHO classification
López-Ginés et al. 2005 [19]	Spain	Retrospective cohort	47	EGFR; chromosome 7 and 10 alterations	Historical histologically defined glioblastoma cohort; IDH not reported; pre-integrated WHO classification
Hobbs et al. 2012 [17]	USA	Retrospective cohort	532	EGFR; MGMT; 10q LOH	Historical molecular pathology glioblastoma cohort; IDH not reported; stratified by EGFR amplification degree
Armocida et al. 2020 [15]	Italy	Retrospective cohort	146	EGFR; IDH	IDH-wildtype glioblastoma cohort
Alimohamadi et al. 2024 [14]	Iran	Prospective cohort	71 adult grade 4 gliomas	EGFR; IDH; TERT; MGMT	Mixed adult grade 4 glioma source cohort: 71 adult grade 4 gliomas: 31 glioblastomas, IDH-wildtype, and 40 astrocytomas, IDH-mutant
Hekimoglu et al. 2024 [16]	Turkey and USA	Retrospective cohort	41 IDH-wildtype butterfly glioblastomas (723 glioblastomas screened)	EGFR; IDH; TERT	IDH-wildtype butterfly glioblastoma cohort; prior diagnosis or treatment of a lower-grade glial tumor excluded
Pulcini et al. 2025 [20]	France	Retrospective cohort	73	EGFR alterations; IDH; MGMT	WHO CNS5 histomolecularly confirmed glioblastoma cohort; astrocytoma, IDH-mutant, CNS WHO grade 4 excluded

Risk of Bias and Study Quality

**Figure 2:**
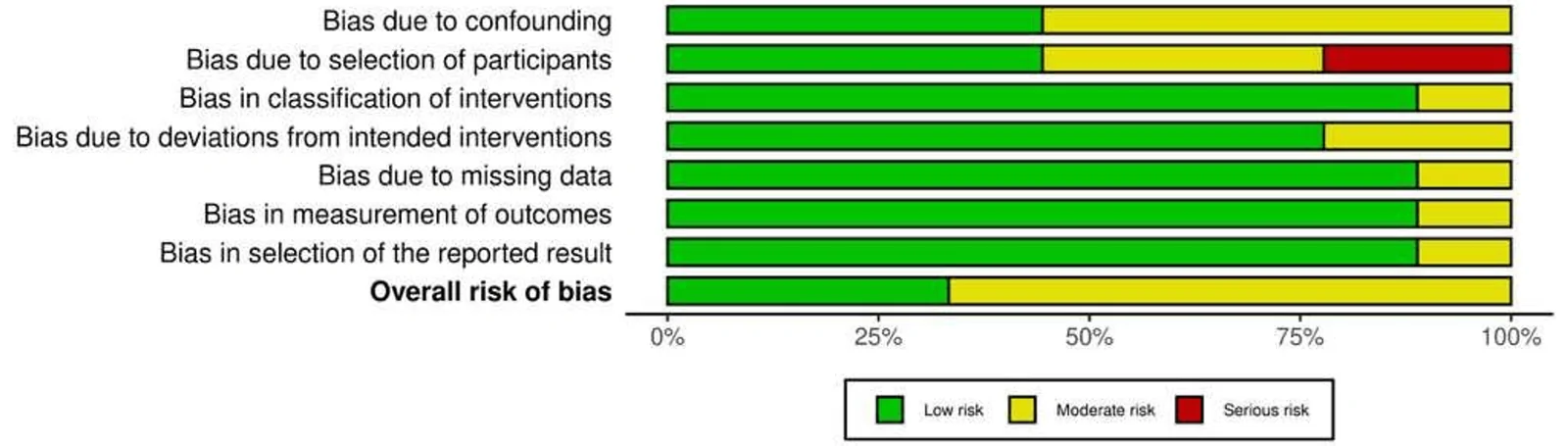
Risk of bias in the included studies as per ROBINS-I assessment tool, including the bias domains

**Figure 3:**
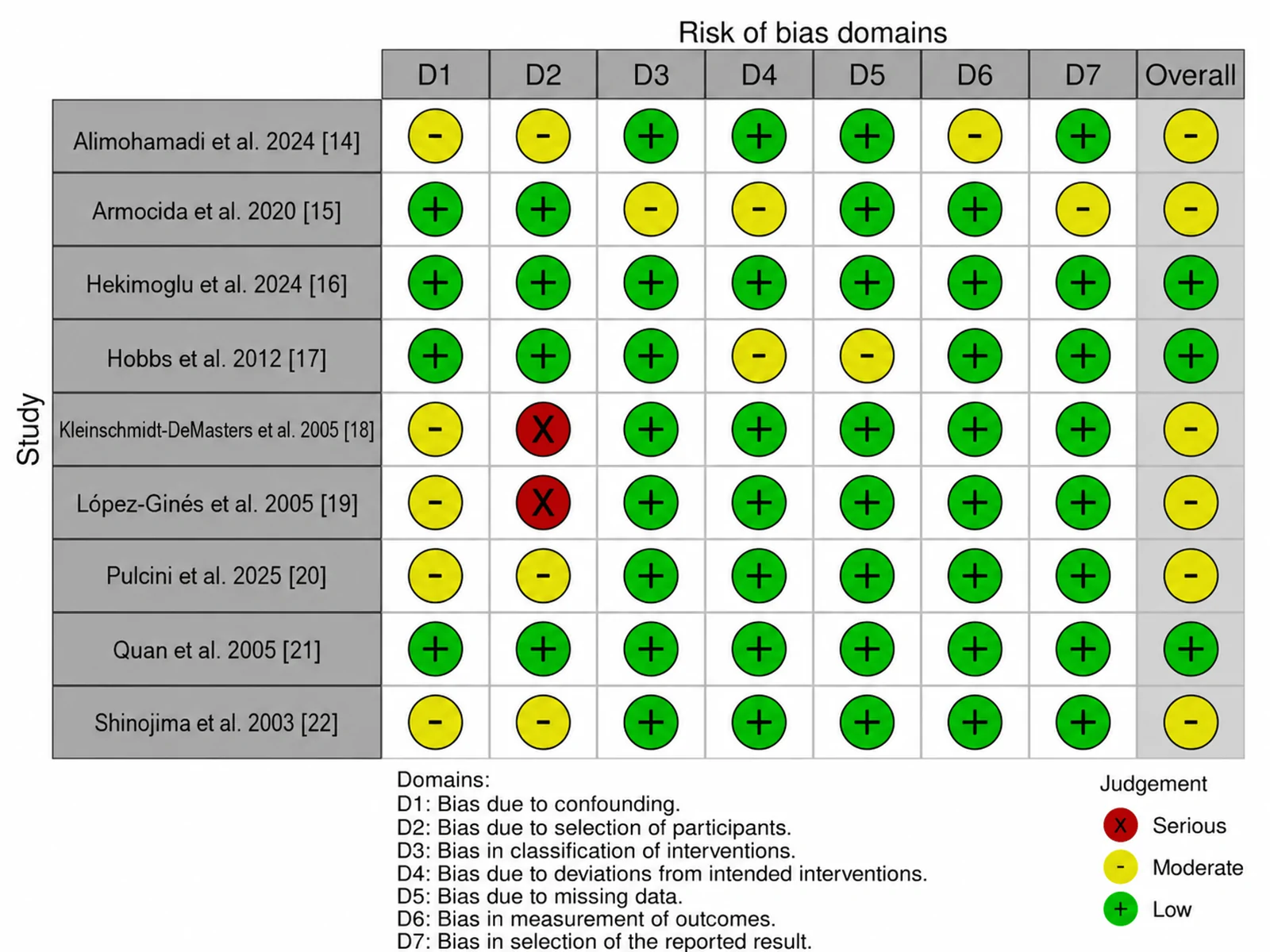
Risk of bias traffic light plot summary

Overall ROBINS-I judgments indicated low risk of bias for three studies (33%) and moderate risk of bias for six studies (67%); no study was judged to have serious or critical overall risk of bias. Domain-level serious concerns were identified only for participant selection in two historical cohorts. Across the included studies, confounding and participant selection were the domains most frequently judged as having moderate risk of bias, whereas exposure classification, missing data, outcome measurement, and selective reporting were predominantly judged to have low risk of bias (Figures 2-3). These findings supported cautious interpretation of pooled estimates, particularly for historical cohorts with greater susceptibility to confounding and participant selection bias.

Newcastle-Ottawa Scale Assessment

Three studies (33%) were rated as high quality and six (67%) as moderate quality according to the Newcastle-Ottawa Scale; no study was rated as low quality (Table 3).

**Table 3:** Quality assessment of the included studies as per the Newcastle-Ottawa Scale

Study	Representativeness of sample	Size sample	Source of information	Demonstration that outcome was not present at study start	Confusion variable control	Assessment of outcome	Enough follow-up period	Newcastle-Ottawa Scale score
Shinojima et al. 2003 [22]	★	★	★	★	★	★	★	7/9 moderate
Kleinschmidt-DeMasters et al. 2005 [18]	★		★	★	★	★	★	6/9 moderate
López-Ginés et al. 2005 [19]	★		★	★	★	★	★	7/9 moderate
Quan et al. 2005 [21]	★★	★★	★	★	★	★	★	9/9 high
Hobbs et al. 2012 [17]	★★	★★	★			★	★	7/9 moderate
Armocida et al. 2020 [15]	★★	★★		★	★	★	★	8/9 high
Hekimoglu et al. 2024 [16]	★★	★★	★	★	★	★	★	9/9 high
Alimohamadi et al. 2024 [14]	★	★	★	★	★	★	★	7/9 moderate
Pulcini et al. 2025 [20]	★	★	★	★	★	★	★	7/9 moderate

Small-Study Effects and Publication Bias

Each pooled comparison included fewer than 10 studies. Therefore, funnel plot asymmetry and Egger's regression were not assessed because these methods have limited power and may yield misleading results when applied to a small number of studies. The absence of formal assessment should not be interpreted as evidence that publication bias or other small-study effects were absent.

Data Synthesis

**Figure 4:**
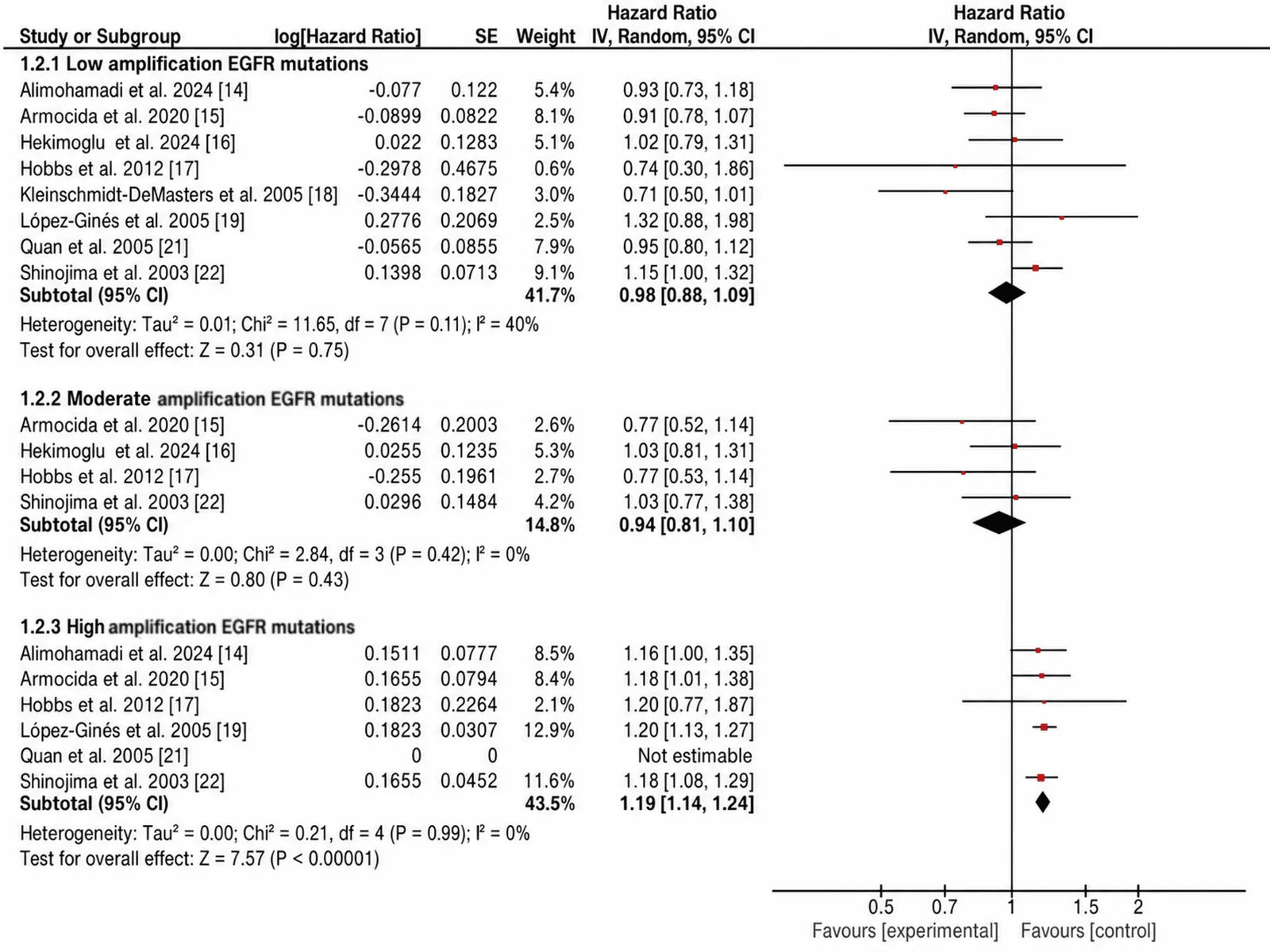
Forest plot showing the relationship among overall survival and EGFR amplification, low amplification, moderate amplification, and high amplification EGFR: epidermal growth factor receptor

OS according to EGFR amplification category: Because definitions of low, moderate, and high EGFR amplification varied across studies, subgroup findings were interpreted according to the categories defined in the individual reports. Figure 4 presents the pooled OS estimates for the low-, moderate-, and high-amplification subgroups.

Low EGFR amplification: Eight studies contributed to the low-amplification subgroup [14-19,21-22]. The pooled HR was 0.98 (95% CI: 0.88-1.09; p=0.75), indicating no statistically significant association with OS. Between-study heterogeneity was moderate (I²=40%).

Moderate EGFR amplification: Four studies contributed to the moderate-amplification subgroup [15-17,22]. The pooled HR was 0.94 (95% CI: 0.81-1.10; p=0.43), indicating no statistically significant association with OS. No between-study heterogeneity was observed (I²=0%).

High EGFR amplification: Six studies were represented in the high-amplification subgroup [14,15,17,19,21,22]. Five contributed estimable effect estimates to the pooled analysis, whereas the comparison reported by Quan et al. was not estimable in the forest plot. The pooled HR was 1.19 (95% CI: 1.14-1.24; p<0.00001), indicating a statistically significant association between high EGFR amplification and poorer OS. No between-study heterogeneity was observed (I²=0%).

Across the included studies, only the high-amplification subgroup demonstrated a statistically significant pooled association with poorer OS, whereas the low- and moderate-amplification subgroups did not. The moderate- and high-amplification subgroups showed no statistical heterogeneity, whereas the low-amplification subgroup demonstrated moderate heterogeneity (I²=40%).

**Figure 5:**
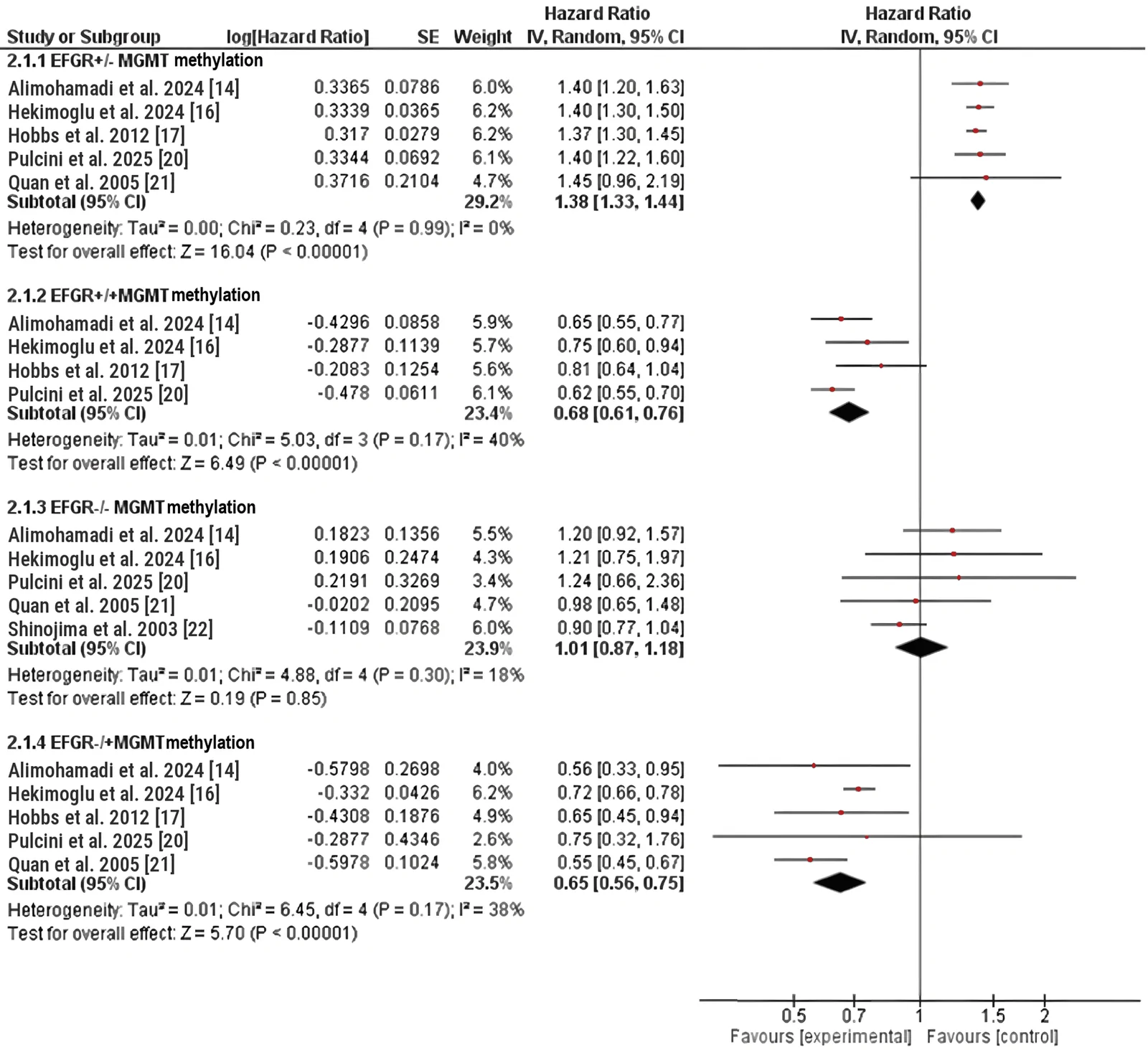
Forest plot depicting the effects of EGFR amplification on survival in glioblastoma patients with MGMT methylation and no MGMT methylation EGFR: epidermal growth factor receptor; MGMT: O6-methylguanine-DNA methyltransferase

OS according to EGFR amplification and MGMT promoter methylation: Figure 5 presents four molecular strata defined by EGFR amplification and MGMT promoter methylation. Because comparator definitions and treatment exposure varied among studies, each pooled estimate is interpreted relative to its study-specific reference group rather than as a direct comparison across all four strata.

Figure 5 presents exploratory pooled estimates for four subgroups defined by EGFR amplification and MGMT promoter methylation status. Because biomarker definitions, reference groups, treatment exposure, assay methods, and covariate adjustment varied across studies, each estimate was interpreted relative to its study-specific comparison and not as a direct comparison among the four molecular subgroups or as evidence of a formal interaction between EGFR amplification and MGMT promoter methylation.

Five studies were represented in the EGFR-positive/MGMT-methylated subgroup [14,16,17,20,21], with a pooled HR of 1.38 (95% CI: 1.33-1.44; p<0.00001; I²=0%).

Four studies were represented in the EGFR-positive/MGMT-unmethylated subgroup [14,16,17,20], with a pooled HR of 0.68 (95% CI: 0.61-0.76; p<0.00001; I²=40%).

Five studies were represented in the EGFR-negative/MGMT-methylated subgroup [14,16,20,22], with a pooled HR of 1.01 (95% CI: 0.87-1.18; p=0.85; I²=18%).

Five studies were represented in the EGFR-negative/MGMT-unmethylated subgroup [14,16-17,20,21], with a pooled HR of 0.65 (95% CI: 0.56-0.75; p<0.00001; I²=38%).

The direction of these exploratory estimates should not be interpreted as demonstrating that MGMT promoter methylation is intrinsically associated with poorer survival, that unmethylated MGMT is protective, or that EGFR amplification and MGMT promoter methylation interact prognostically. Such conclusions would require uniformly defined molecular strata, comparable reference groups, and formal interaction testing.

IDH status across the included cohorts: IDH ascertainment and molecular classification differed substantially across the included studies. Five studies [17-19,21,22] were conducted before contemporary integrated molecular diagnosis and did not provide an eligible EGFR-specific survival estimate within an IDH-defined tumor entity [17-19,21,22]. These cohorts were classified histologically as glioblastoma, and the information reported in the original publications was insufficient to determine whether all tumors would meet current criteria for glioblastoma, IDH-wildtype.

Three contemporary studies evaluated populations restricted to IDH-wildtype disease. Armocida et al. studied patients with IDH-wildtype glioblastoma [15], while Hekimoglu et al. analyzed 41 patients with IDH-wildtype butterfly glioblastoma [16]. Pulcini et al. reviewed all tumors according to WHO CNS5 and explicitly excluded astrocytoma, IDH-mutant, CNS WHO grade 4 [20]. These studies therefore characterize EGFR-related findings within IDH-wildtype glioblastoma but cannot provide evidence regarding EGFR-associated survival in IDH-mutant grade 4 astrocytoma.

Alimohamadi et al. enrolled 71 adult non-H3-altered grade 4 gliomas that were reclassified as 31 glioblastomas, IDH-wildtype, and 40 astrocytomas, IDH-mutant, CNS WHO grade 4 [14]. Although survival differed significantly according to IDH status, the study did not report a directly extractable HR comparing EGFR-amplified and EGFR-non-amplified tumors separately within each IDH-defined entity.

Because comparable EGFR-specific HRs within molecularly defined IDH subgroups were not consistently available, a pooled analysis according to combined EGFR and IDH status was not performed. IDH status was instead used to characterize cohort composition and to inform the interpretation of the pooled EGFR estimates.

Discussion

Principal Findings

This meta-analysis identified different pooled OS estimates across the EGFR amplification categories reported by the included studies. Low- and moderate-amplification categories were not significantly associated with OS, whereas the category classified as high EGFR amplification was associated with a higher mortality hazard (HR: 1.19; 95% CI: 1.14-1.24). Because amplification thresholds and assay methods varied across studies and no formal dose-response or trend analysis was performed, these findings should not be interpreted as establishing a universal biological threshold. Rather, they indicate that the adverse pooled association was confined to the study-defined high-amplification category. The findings also do not establish EGFR amplification as an independent prognostic determinant because adjustment for IDH-defined tumor entity, treatment, age, performance status, extent of resection, and other relevant covariates was inconsistent across cohorts.

The exploratory analyses according to combined EGFR and MGMT status produced pooled estimates that differed in direction across molecular strata. However, the contributing studies varied in comparator definitions, temozolomide exposure, biomarker assays, diagnostic era, and covariate adjustment. The four pooled estimates therefore should not be interpreted as direct comparisons among the molecular strata, as evidence that MGMT promoter methylation is intrinsically adverse, or as proof of a statistical interaction between EGFR amplification and MGMT promoter methylation. Collectively, the findings suggest that the observed association between EGFR amplification and survival is context-dependent, but the available evidence does not permit a definitive quantitative ranking of EGFR/MGMT-defined subgroups.

Molecular Interpretation Within WHO CNS5 and TCGA Frameworks

IDH status defines the relevant tumor entity under WHO CNS5 and is indispensable to interpreting EGFR-associated survival; however, the included studies did not provide sufficiently comparable EGFR-specific estimates to support a quantitative analysis within IDH-defined subgroups. WHO CNS5 restricts the diagnosis of glioblastoma to IDH-wildtype tumors, whereas tumors historically designated as secondary or IDH-mutant glioblastoma are now classified as astrocytoma, IDH-mutant, CNS WHO grade 4 [2-5,23-25]. EGFR amplification is enriched in glioblastoma, IDH-wildtype, and is uncommon in IDH-mutant diffuse gliomas [3-5,25]. Accordingly, EGFR and IDH should not be treated as independent biomarkers randomly distributed within one disease population.

The included evidence spans substantially different diagnostic eras. Several historical studies classified tumors according to histopathologic criteria without routine IDH testing, whereas contemporary cohorts were restricted to molecularly defined IDH-wildtype glioblastoma or explicitly excluded astrocytoma, IDH-mutant, CNS WHO grade 4. Consequently, an apparent association between EGFR amplification and poorer survival in the pooled historical literature may partly reflect differences in underlying tumor composition rather than an isolated effect of EGFR. This limitation is illustrated by Alimohamadi et al., in which 71 tumors initially diagnosed histologically as glioblastoma were reclassified into 31 glioblastomas, IDH-wildtype, and 40 astrocytomas, IDH-mutant; the two entities had significantly different survival outcomes [14].

TCGA-based molecular studies provide complementary biological context by demonstrating substantial genomic heterogeneity within diffuse gliomas and enrichment of EGFR alterations within specific molecular patterns. Nevertheless, TCGA transcriptional or genomic subtypes should not be equated directly with WHO CNS5 diagnostic entities. WHO classification establishes the diagnosis, whereas TCGA-derived profiles help characterize the biological heterogeneity that remains within those entities.

From a biological perspective, EGFR-driven signaling operates within a heterogeneous and frequently hypoxic tumor ecosystem in which autophagy and clonal interactions can influence invasion and treatment response [26-28]. EGFR, also known as ERBB1 or HER1, is a receptor tyrosine kinase whose amplification can increase receptor signaling through proliferative and survival pathways, including PI3K-AKT-mTOR and RAS-RAF-MEK-ERK signaling [29-33]. Experimental studies of autophagy and EGFR modulation support effects on migration, invasion, and radiosensitivity [34-36], while broader phosphoproteomic work has characterized the oncogenic EGFR signaling network [37]. EGFRvIII and related expression patterns add another layer of molecular heterogeneity, although these alterations are biologically related to, but not interchangeable with, EGFR gene amplification [38,39]. The prevalence and interpretation of EGFR alterations also vary across the historically described primary and secondary glioblastoma pathways and across histologic variants such as small cell glioblastoma, gliosarcoma, and giant cell glioblastoma [40-46]. These mechanisms establish biological plausibility for an association between EGFR alterations and tumor behavior, but they do not independently establish the prognostic effect of EGFR amplification or eliminate the need to account for IDH-defined tumor entity, treatment exposure, and relevant clinical covariates when estimating prognosis [45-47].

MGMT promoter methylation represents a different biological and clinical process. MGMT encodes a DNA repair enzyme that removes alkyl lesions from the O6 position of guanine; promoter methylation reduces MGMT expression and is associated with greater benefit from temozolomide-based treatment [47-49]. Its relationship with observed survival therefore depends on alkylating agent exposure, assay methodology, methylation cutoff, and the molecular composition of the cohort. IDH-mutant gliomas additionally exhibit widespread epigenetic remodeling, including the glioma CpG island methylator phenotype, further limiting direct comparison with IDH-wildtype glioblastoma [50].

Accordingly, EGFR and MGMT should not be interpreted as equivalent or simply additive prognostic markers. EGFR amplification reflects proliferative signaling within an IDH-defined tumor entity, whereas MGMT promoter methylation principally informs DNA repair capacity and expected sensitivity to temozolomide. The combined EGFR/MGMT estimates in this review should therefore be regarded as exploratory observations generated at the intersection of tumor biology, treatment responsiveness, diagnostic era, and study-specific comparator definitions.

Prognostic and Clinical Implications

The statistically significant pooled association observed in the study-defined high-amplification category is hypothesis-generating. Because EGFR amplification was measured using different assays and thresholds, and because adjustment for age, performance status, extent of resection, treatment, IDH-defined tumor entity, and other established prognostic factors was inconsistent, the result does not establish a universal biological threshold or an independent prognostic effect. The available evidence does not support altering surgical, radiotherapeutic, systemic treatment, surveillance, or counseling decisions according to EGFR amplification alone. EGFR amplification should instead be considered a complementary molecular feature whose prognostic value remains dependent on tumor classification and clinical context.

IDH status remains essential because it defines the underlying adult diffuse glioma entity under WHO CNS5. The included studies did not provide sufficiently comparable EGFR-specific estimates within IDH-defined entities to determine whether EGFR amplification modifies survival differently in glioblastoma, IDH-wildtype, and astrocytoma, IDH-mutant, CNS WHO grade 4. Historical histologically defined cohorts, contemporary IDH-wildtype cohorts, and mixed grade 4 glioma populations should therefore not be interpreted as biologically interchangeable.

MGMT promoter methylation should be interpreted separately from EGFR amplification. It reflects reduced DNA repair capacity and is associated with greater benefit from temozolomide-based therapy, although its observed relationship with outcome depends on treatment exposure, testing method, and methylation cutoff. Contemporary consensus literature does not support EGFR amplification as a consistently established outcome marker in glioblastoma, whereas MGMT methylation has demonstrated treatment relevance in temozolomide-treated populations.

The pooled EGFR/MGMT findings should not be interpreted as evidence that MGMT promoter methylation is harmful, that unmethylated MGMT is protective, or that EGFR and MGMT interact prognostically. Such conclusions require consistently defined joint molecular strata, a common or clearly harmonized comparator, and a formal interaction analysis. Given the heterogeneity in molecular definitions, reference groups, treatment exposure, assay methods, and covariate adjustment, these exploratory estimates should not currently inform clinical stratification.

Limitations

The included evidence spanned substantially different diagnostic, treatment, and molecular classification eras. Several historical cohorts were classified according to histopathologic criteria before routine IDH testing and integrated molecular diagnosis. Consequently, some tumors historically designated as glioblastoma may correspond under WHO CNS5 to astrocytoma, IDH-mutant, CNS WHO grade 4. In contrast, contemporary studies generally evaluated molecularly defined glioblastoma, IDH-wildtype. These differences limit direct comparability between historical and contemporary cohorts.

One prospective study analyzed a mixed cohort of 71 adult grade 4 gliomas, including 31 glioblastomas, IDH-wildtype, and 40 astrocytomas, IDH-mutant, CNS WHO grade 4, and did not report EGFR-specific survival estimates separately within each entity. Because the reported estimate reflected the mixed cohort, its contribution to the quantitative synthesis cannot be interpreted as a glioblastoma-specific effect and limits entity-level inference.

EGFR amplification was assessed using different molecular platforms, amplification thresholds, and comparison categories. The low-, moderate-, and high-amplification categories were defined by the individual studies and should not be assumed to represent standardized or biologically equivalent exposure levels. Some reports also evaluated broader EGFR alterations or related measures that were not interchangeable with EGFR gene amplification. No formal dose-response or trend analysis was performed.

The contributing studies differed in age, performance status, extent of resection, treatment era, temozolomide exposure, follow-up duration, and covariate adjustment. Several studies evaluated selected clinical populations, including elderly patients and patients with butterfly glioblastoma, which may limit generalizability to unselected contemporary glioblastoma populations. In addition, the degree of statistical adjustment varied across reports; therefore, the pooled estimates should not be interpreted as uniformly adjusted independent prognostic effects.

The combined EGFR/MGMT analyses were limited by differences in biomarker definitions, MGMT testing methods and cutoffs, treatment exposure, reference groups, and covariate adjustment. These estimates were exploratory and did not constitute direct comparisons among the molecular strata or formal tests of interaction between EGFR amplification and MGMT promoter methylation.

Eight of the nine included studies were retrospective, increasing susceptibility to participant selection bias and confounding. Random-effects models allowed for statistical between-study variation but could not eliminate differences in population composition, exposure classification, comparator definition, or study design. The small number of studies within each analysis precluded reliable meta-regression and formal assessment of funnel plot asymmetry. Multiple sparse subgroup analyses also increased the possibility of chance findings, and statistical significance within one subgroup together with nonsignificance in another does not establish effect modification.

Publication bias and selective outcome reporting cannot be excluded. Accordingly, the pooled findings should be interpreted as hypothesis-generating and require confirmation in prospective WHO CNS5-defined glioblastoma cohorts using standardized EGFR assays, prespecified amplification thresholds, uniform comparator definitions, detailed treatment reporting, and adequately adjusted multivariable models.

Strengths and Future Directions

This review brings together foundational and contemporary evidence and interprets the findings in the context of the transition from histologic diagnosis to integrated molecular classification. Additional strengths include independent study selection and data extraction, complementary assessment using ROBINS-I and the Newcastle-Ottawa Scale, consideration of diagnostic era and assay methodology, and cautious interpretation of exploratory subgroup analyses.

Future prognostic studies should enroll molecularly defined glioblastoma, IDH-wildtype, populations and report the EGFR copy-number assay, amplification threshold, and distribution of amplification values transparently. Studies should also document MGMT testing methodology and cutoff, temozolomide exposure, age, performance status, extent of resection, treatment characteristics, and other clinically relevant covariates and should report adjusted HRs with corresponding measures of precision.

When studies include more than one adult grade 4 glioma entity, analyses should be stratified according to WHO CNS5 diagnosis rather than treating IDH status as an additional covariate within a biologically mixed population. Adequately powered studies using standardized joint biomarker definitions and formal interaction terms are needed to determine whether EGFR amplification contributes prognostic information beyond established clinical factors and MGMT promoter methylation within glioblastoma, IDH-wildtype. A larger and more harmonized evidence base would also permit more reliable meta-regression and formal evaluation of effect modification.

## Conclusions

Across the EGFR amplification categories reported by the included studies, low and moderate amplification were not significantly associated with OS, whereas the study-defined high-amplification category demonstrated a statistically significant association with poorer OS. Because amplification thresholds, assay methods, diagnostic eras, treatment exposure, and covariate adjustment varied across studies, this finding should be regarded as hypothesis-generating and should not be interpreted as establishing a universal biological threshold or an independent prognostic effect.

EGFR amplification should not be considered an isolated determinant of outcome. Under WHO CNS5, IDH status defines the tumor entity and the primary molecular context in which EGFR-associated findings must be interpreted. MGMT promoter methylation is an established treatment-relevant marker associated with expected benefit from temozolomide-based therapy, whereas EGFR amplification remains biologically important but inconsistently associated with prognosis. Contemporary consensus literature similarly supports MGMT as treatment-relevant while not recognizing EGFR amplification as a consistently established outcome marker.

The observed pooled associations may reflect EGFR-related tumor biology together with differences in IDH-defined cohort composition, diagnostic era, treatment, and molecular testing methodology. Prospective studies using WHO CNS5-defined glioblastoma, standardized EGFR assays and amplification thresholds, transparent comparator definitions, and adequately adjusted multivariable models are required before EGFR amplification can be regarded as an independent prognostic biomarker or incorporated into clinical risk stratification models.

## Appendices

Search strategy

PubMed/MEDLINE

(Glioblastoma OR "glioblastoma multiforme" OR GBM OR "brain cancer" OR "malignant glioma") AND ("Receptor, Epidermal Growth Factor"[MeSH Terms] OR "Gene Amplification"[MeSH Terms] OR "EGFR amplification" OR "epidermal growth factor receptor amplification" OR "EGFR gene amplification" OR "EGFR copy number gain" OR "EGFR overexpression") AND ("Survival Analysis"[MeSH Terms] OR "Overall Survival"[MeSH Terms] OR survival OR "overall survival" OR prognosis OR mortality OR "survival rate" OR outcome)

Cochrane Library

(glioblastom* OR gbm* OR glioma* malignan* OR "brain cancer") AND (EGFR amplific* OR "epidermal growth factor receptor amplific*" OR "EGFR gene amplific*" OR "EGFR copy number gain*" OR EGFR overexpress*) AND (surviv* OR "overall survival" OR prognos* OR mortality)

Scopus

( TITLE-ABS-KEY ( "brain cancer" OR glioblastom* OR gbm* OR ( glioma* AND malignan* ) ) ) AND ( TITLE-ABS-KEY ( "EGFR amplification" OR "epidermal growth factor receptor amplification" OR "EGFR gene amplification" OR "EGFR copy number gain" OR "EGFR overexpression" OR EGFR amplific* ) ) AND ( TITLE-ABS-KEY ( surviv* OR "overall survival" OR prognos* OR mortality ) ) AND ( PUBYEAR > 2018 ) AND (DOCTYPE ( ar OR re ) )

Embase

1.(glioblastom* OR gbm* OR (glioma* AND malignan*) OR "brain cancer" OR 'glioblastoma'/exp)

2.("EGFR amplification" OR "epidermal growth factor receptor amplification" OR "EGFR gene amplification" OR "EGFR copy number gain" OR "EGFR overexpression" OR EGFR amplific* OR 'epidermal growth factor receptor gene amplification'/exp)

3. (surviv* OR "overall survival" OR prognos* OR mortality OR 'survival analysis'/exp)

1 AND 2 AND 3

CINAHL

(MH "Glioblastoma" OR glioblastom* OR gbm* OR (glioma* AND malignan*) OR "brain cancer") AND (MH "Epidermal Growth Factor Receptor" OR "EGFR amplification" OR "epidermal growth factor receptor amplification" OR "EGFR gene amplification" OR "EGFR copy number gain" OR "EGFR overexpression" OR EGFR amplific*) AND (MH "Survival Rate" OR surviv* OR "overall survival" OR prognos* OR mortality)

Web of Science

(glioblastom* OR gbm* OR (glioma* AND malignan*) OR "brain cancer") AND ("EGFR amplification" OR "epidermal growth factor receptor amplification" OR "EGFR gene amplification" OR "EGFR copy number gain" OR "EGFR overexpression" OR EGFR amplific*) AND (surviv* OR "overall survival" OR prognos* OR mortality)
